# Commentary: Sensory integration dynamics in a hierarchical network explains choice probabilities in cortical area MT

**DOI:** 10.3389/fnsys.2016.00037

**Published:** 2016-05-03

**Authors:** Anne E. Urai, Peter R. Murphy

**Affiliations:** ^1^Department of Neurophysiology and Pathophysiology, University Medical Center Hamburg-EppendorfHamburg, Germany; ^2^Department of Psychology, University of AmsterdamAmsterdam, Netherlands

**Keywords:** perceptual decision-making, biophysical modeling, motion discrimination, top-down feedback, psychophysical reverse-correlation

The phenomenon of choice probability was first described by Britten et al. (1996), who measured firing rates of neurons in area MT that are highly sensitive to visual motion. In this influential study, when a macaque monkey viewed a cloud of moving dots in an effort to determine its dominant direction of motion, the firing rates of single MT neurons were found to predict the decision that the monkey would eventually make. Interestingly, this was the case even when identical stimuli were presented multiple times, showing that sensory neurons carry information about the monkey's decision beyond that which is present in the stimulus. Choice probability, which quantifies how well the monkey's choices can be predicted from such neural activity, thus reflects a decision-maker's variable, subjective judgments about sensory information in the outside world (Crapse and Basso, 2015).

How can neurons at this early stage of the visual processing hierarchy “know” about the final decision the monkey will make? Responses of neurons in sensory regions like area MT are highly variable from trial to trial even when evoked by the same stimulus, while higher-level areas involved in the decision-making process “read-out” this noisy information to form a choice. In a strictly bottom-up view of choice probability, the phenomenon arises because fluctuations in the firing of MT neurons causally contribute to the decision that is ultimately made (Shadlen et al., 1996). That is, if the firing rates of neurons in an MT population fluctuate randomly, but together over trials (a commonly observed phenomenon termed noise correlation that likely has multiple origins), this common activation cannot be “averaged out” by downstream decision neurons and, in turn, will influence choice.

However, low-level visual neurons also receive dense feedback projections from upstream regions involved in decision-making. Another explanation of choice probability is thus that the dynamics of decision formation in high-level association cortex shape activity in visual cortex through feedback. In this top-down scenario, choice probability in areas like MT does not reflect a causal influence on the decision, but rather results from the decision process that takes place further up the cortical hierarchy. Consistent with such an account, Nienborg and Cumming (2009) found that the time course of choice probability in macaque area V2 rose quickly upon stimulus onset and then plateaued over time. This sustained temporal profile is consistent with the choice probabilities observed in area MT (Britten et al., 1996). On the other hand, the “psychophysical kernel”—showing when fluctuations in the stimulus influence behavior the most—was observed to peak early and decrease as the trial unfolded. Thus, although neural activity in sensory cortex remained predictive of the upcoming choice toward the end of the trial, the information in the stimulus had little impact on choice at this time. This dissociation indicates that choice probability reflects more than a bottom-up influence of sensory information on behavior.

Now, a recent paper by Wimmer et al. (2015) has employed sophisticated computational modeling and novel analyses of existing empirical data in an effort to dissociate bottom-up and top-down influences on choice probability. The authors extended an existing neurobiologically principled model of decision-making (Wang, 2002), which replicates essential features of firing rates in cortical area LIP that are thought to track decision formation. Specifically, they incorporated populations of sensory neurons (representing area MT) that relay stimulus information to the decision circuits of the original model and, critically, receive feedback connections from these circuits (Figure 1A).

**Figure 1:**
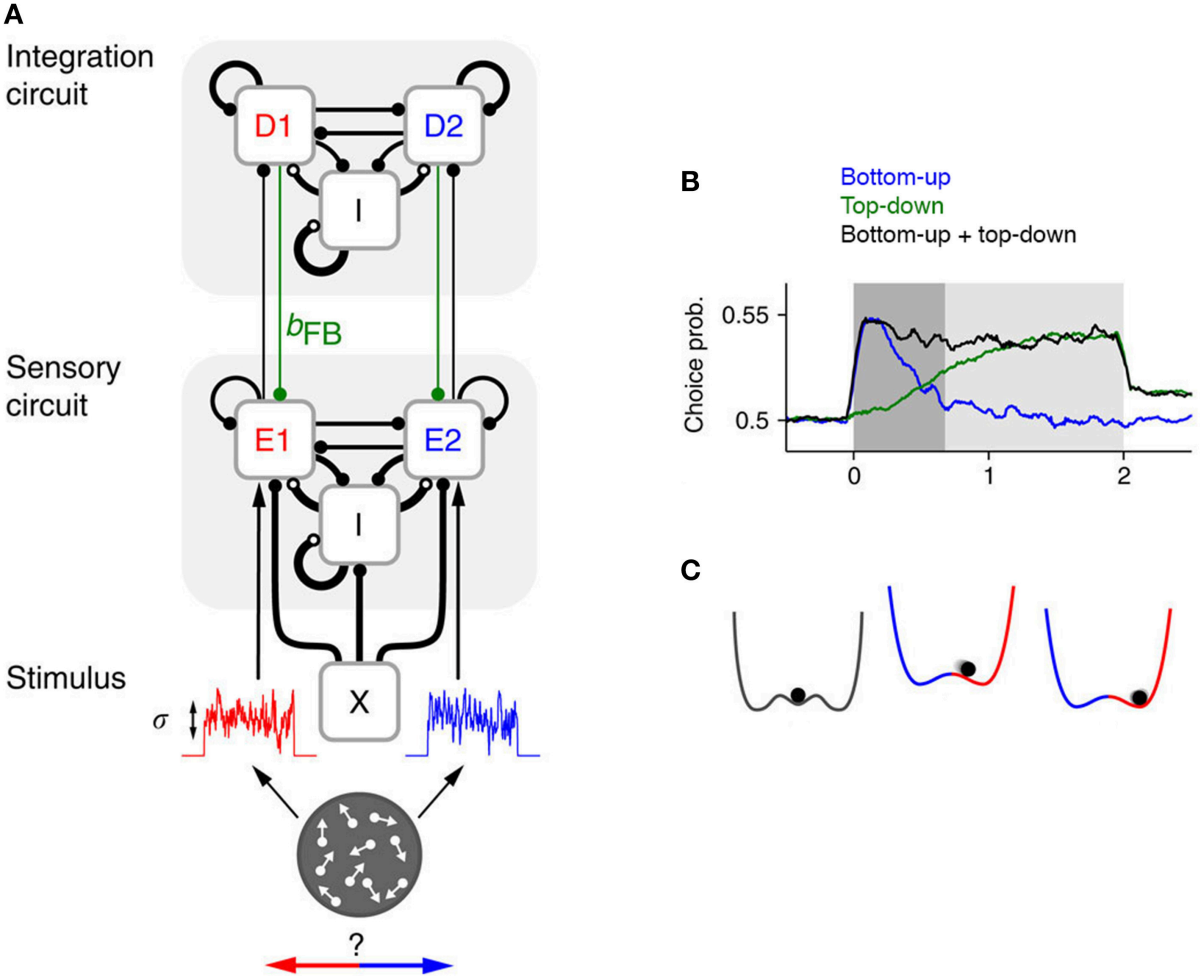
**(A)** Graphical representation of the hierarchical network model constructed by Wimmer et al. (2015), which contains a layer of sensory neurons (with red and blue denoting populations selective for choice “left” and “right”, respectively) and a layer of integrator or decision neurons. The strength of the feedback from decision to sensory regions (in green), which is mediated by fast AMPA receptors in the model, can be systematically varied to assess the effect of top-down signals on choice probability and behavior. **(B)** A combination of early bottom-up and late top-down influences leads to a sustained choice probability signal, as observed in neural recordings. **(C)** Commitment to a choice corresponds to a well in the energy-landscape of the decision process. A well represents a stable state, which is maintained unless a lot of additional input is added to a system to shift it out of that state. All panels are adapted from Wimmer et al. (2015) under a CC-BY license.

By simulating data from models in which the relative dominance of bottom-up and top-down components was systematically varied, Wimmer et al.'s analysis suggests that the empirically observed pattern of sustained choice probability likely arises from a combination of both factors (Figure 1B). Early in the trial, bottom-up fluctuations in the firing of MT neurons exert a causal influence on choice and lead to a fast-rising choice probability. Later in the trial, top-down influences take over. The model also generated novel predictions about the ways in which bottom-up and top-down factors should affect both the temporal stability of choice probabilities and the structure of the correlations between pairs of MT neurons. The authors verified that, remarkably, each of the predicted patterns is present in the original electrophysiological recordings from Britten et al. (1996).

Furthermore, by analysing the psychophysical kernels of new monkeys performing the motion discrimination task, Wimmer et al. corroborated previous observations (Kiani et al., 2008; Nienborg and Cumming, 2009) that monkeys tend to commit to their decisions relatively early in the trial and disregard subsequent sensory evidence. This “primacy effect” is a natural consequence of a neural network architecture that, like the original model that Wimmer et al. extend, encourages competitive, winner-take-all “attractor” dynamics (Figure 1C). By incorporating feedback connections into this model, Wimmer et al. neatly reconcile this effect with the sustained choice probabilities observed in empirical data.

Beyond this elegant account of choice probability, incorporating feedback connections into their model also allowed the authors to make several novel observations. For example, they detail how top-down feedback connections first serve to increase the rate at which the competition between alternative choices is resolved, and subsequently reinforce the initially winning choice at the expense of a more protracted and accurate decision process. This feedback-led acceleration of decision dynamics may point to a novel, biologically plausible role for feedback connections in determining the speed-accuracy trade-off, and leads to the prediction that individuals with stronger feedback connections should exhibit stronger primacy and a greater emphasis on speed over accuracy.

Wimmer et al. also note that the feedback-accelerated choice resolution implies that feedback connections might influence decision confidence: when feedback is strong, the difference in “neural evidence” between choice alternatives tends to be high, which in turn might equate to higher confidence in the final choice. Coupled with the possible role of feedback connections in the emphasis of speed over accuracy, this reasoning suggests that, in some settings at least, faster and less accurate decision-making should be accompanied by greater confidence. This is surprising, since accuracy and confidence are typically observed to be positively correlated. It will be interesting to see whether future studies that measure or manipulate the strength of top-down feedback connections can find support for this prediction.

Lastly, the modeling approach used by Wimmer et al. offers great scope for bridging across different levels of analysis and revealing the mechanistic significance of physiological signals that are measurable in human subjects. Such models are particularly appealing in this regard because they leverage biophysical principles to simulate actual neuronal spiking data, which can then be averaged within and across populations of neurons to derive predictions about what might be observed at coarser spatial scales. For example, it should be possible to calculate measures of the interaction or coherence between distinct neuronal populations in the model and investigate equivalent signals using human scalp electrophysiology (M/EEG), a field in which analysis of inter-areal coherence or information transfer is commonplace. Such an approach holds obvious promise for augmenting our understanding of the neural dynamics underlying animal and human decision-making, in both health and disease.

## Author contributions

All authors listed, have made substantial, direct and intellectual contribution to the work, and approved it for publication.

### Conflict of interest statement

The authors declare that the research was conducted in the absence of any commercial or financial relationships that could be construed as a potential conflict of interest.
